# Assessment of cerebral autoregulatory function and inter-hemispheric blood flow in older adults with internal carotid artery stenosis using transcranial Doppler sonography-based measurement of transient hyperemic response after carotid artery compression

**DOI:** 10.1007/s11357-023-00896-1

**Published:** 2023-08-21

**Authors:** Rita Magyar-Stang, Hanga Pál, Borbála Csányi, Anna Gaál, Zsuzsanna Mihály, Zsófia Czinege, Tamas Csipo, Zoltan Ungvari, Péter Sótonyi, Andrea Varga, Tamás Horváth, Dániel Bereczki, Akos Koller, Róbert Debreczeni

**Affiliations:** 1 Department of Neurology, Semmelweis University, Budapest, Hungary; 2 János Szentágothai Doctoral School of Neurosciences, Semmelweis University, Budapest, Hungary; 3 Faculty of Medicine, Semmelweis University, Budapest, Hungary; 4 Department of Vascular and Endovascular Surgery, Semmelweis University, Budapest, Hungary; 5 Vascular Cognitive Impairment and Neurodegeneration Program, Oklahoma Center for Geroscience and Healthy Brain Aging, Department of Neurosurgery, University of Oklahoma Health Sciences Center, Oklahoma City, OK 731042 USA; 6 International Training Program in Geroscience, Doctoral School of Basic and Translational Medicine/Department of Public Health, Semmelweis University, Budapest, Hungary; 7 Peggy and Charles Stephenson Cancer Center, Oklahoma City, OK 73104 USA; 8 Department of Health Promotion Sciences, College of Public Health, University of Oklahoma Health Sciences Center, Oklahoma City, OK USA; 9 Department of Diagnostic Radiology, Heart and Vascular Center, Semmelweis University, Budapest, Hungary; 10 Research Center for Sport Physiology, Hungarian University of Sports Science, Budapest, Hungary; 11 Department of Morphology & Physiology, Faculty of Health Sciences, and Translational Medicine Institute, Faculty of Medicine, and ELKH-SE, Cerebrovascular and Neurocognitive Disorders Research Group, Semmelweis University, Budapest, Hungary; 12 Department of Physiology, New York Medical College, Valhalla, NY USA

**Keywords:** Cerebrovascular reactivity, Transcranial Doppler sonography, Carotid artery stenosis, Transient hyperemic response, Cerebral hemodynamics

## Abstract

Unhealthy vascular aging promotes atherogenesis, which may lead to significant internal carotid artery stenosis (CAS) in 5 to 7.5% of older adults. The pathogenic factors that promote accelerated vascular aging and CAS also affect the downstream portion of the cerebral microcirculation in these patients. Primary treatments of significant CAS are eversion endarterectomy or endarterectomy with patch plasty. Factors that determine adequate hemodynamic compensation and thereby the clinical consequences of CAS as well as medical and surgical complications of carotid reconstruction surgery likely involve the anatomy of the circle of Willis (CoW), the magnitude of compensatory inter-hemispheric blood flow, and the effectiveness of cerebral microcirculatory blood flow autoregulation. This study aimed to test two hypotheses based on this theory. First, we hypothesized that patients with symptomatic and asymptomatic CAS would exhibit differences in autoregulatory function and inter-hemispheric blood flow. Second, we predicted that anatomically compromised CoW would associate with impaired inter-hemispheric blood flow compensation. We enrolled older adults with symptomatic or asymptomatic internal CAS (>70% NASCET criteria; *n* = 46) and assessed CoW integrity by CT angiography. We evaluated transient hyperemic responses in the middle cerebral arteries (MCA) after common carotid artery compression (CCC; 10 s) by transcranial Doppler sonography (TCD). We compared parameters reflecting autoregulatory function (e.g., transient hyperemic response ratio [THRR], return to baseline time [RTB], changes of vascular resistance) and inter-hemispheric blood flow (residual blood flow velocity). Our findings revealed that CAS was associated with impaired cerebral vascular reactivity. However, we did not observe significant differences in autoregulatory function or inter-hemispheric blood flow between patients with symptomatic and asymptomatic CAS. Moreover, anatomically compromised CoW did not significantly affect these parameters. Notably, we observed an inverse correlation between RTB and THRR, and 49% of CAS patients exhibited a delayed THRR, which associated with decreased inter-hemispheric blood flow. Future studies should investigate how TCD-based evaluation of autoregulatory function and inter-hemispheric blood flow can be used to optimize surgical techniques and patient selection for internal carotid artery revascularization.

## Introduction

Internal carotid artery stenosis (CAS) is a steno-occlusive arterial disease, usually caused by atherosclerosis associated with unhealthy vascular aging [1] The prevalence of CAS is approximately 5 to 7.5% in older adults [2–4] There is significant variability in the severity of clinical manifestations of CAS. The atherosclerotic plaque causing the narrowing of the internal carotid artery can be stable and asymptomatic, or it can be a source of thromboembolization to the brain, leading to ischemic events (“symptomatic CAS,” approximately one-third of all CAS cases [5]). Symptomatic CAS is characterized by either symptoms associated with temporary brain ischemia (transient ischemic attack [TIA]) or ischemic stroke. Atherosclerotic CAS is a leading risk factor for ischemic stroke, which is a major cause of mortality and long-term disability in developed countries [6–8]. To prevent stroke, eligible patients with significant CAS are treated with either eversion endarterectomy or endarterectomy with patch plasty [1, 9].

The mechanisms by which CAS promotes cerebral ischemic events are thought to involve hemodynamic mechanisms in addition to thromboembolism [10–16]. It has also been proposed that dysregulation of cerebral blood flow regulation in CAS patients may also contribute to perioperative complications during carotid endarterectomy or carotid artery reconstruction surgery [5, 17, 18]. Factors that determine adequate hemodynamic compensation and thereby the clinical consequences of CAS as well as medical and surgical complications of carotid reconstruction surgery likely involve the anatomy of the circle of Willis (CoW) [13, 19–31], the magnitude of compensatory inter-hemispheric blood flow, and the effectiveness of cerebral microcirculatory blood flow autoregulation [10–16, 19, 20, 32–38].

The pathogenic factors that accelerate vascular aging, exacerbate atherogenesis in the large arteries, and promote the genesis of CAS also affect the function and phenotype of resistance arteries in the brain [39–47]. It has been proposed that the resulting functional impairment of microvessels may compromise cerebral blood flow regulation [10–16, 19, 20, 32–38] and exacerbate the deleterious effects of thromboembolism, increasing the risk of infarction in the border zone areas of the brain [48–55]. Autoregulation of cerebral blood flow is a homeostatic mechanism that is critical for the maintenance of oxygen and nutrient supply to the brain when perfusion pressure decreases [16, 39, 56–59]. Importantly, CAS has been linked to autoregulatory dysfunction in older patients [11, 14–16, 37]. Yet, the differential effects of symptomatic and asymptomatic CAS on autoregulation of cerebral blood flow remain elusive.

A well-developed collateral circulation is critical for the maintenance of cerebral perfusion through inter-hemispheric blood flow compensation in patients with CAS [10]. Impairment of inter-hemispheric blood flow due to anatomical or functional alterations of the collateral circulation increases the risk of stroke and exacerbates perioperative complications in these patients [18, 27, 60]. Among the factors contributing to impaired inter-hemispheric blood flow compensation in CAS, decreased cerebral primary anastomosis capacity based on abnormal anatomical variations of the CoW [21–31] communicating connections plays a central role [61, 62]. The prevalence of CoW variations is unexpectedly high [63]. It is presently unknown how absence of collateral flow via the CoW and impaired vasomotor reactivity are related.

The present study was conducted to test two related hypotheses: (1) that patients with symptomatic and asymptomatic CAS exhibit differences in autoregulatory function and inter-hemispheric blood flow, and (2) that anatomically compromised CoW results in impaired inter-hemispheric blood flow compensation. To test these predictions, in older adults with symptomatic or asymptomatic internal CAS (>70%), CoW integrity was assessed by CT angiography and cerebral vasoreactivity and autoregulatory function was assessed by a transcranial Doppler sonography (TCD)-based method.

TCD is one of the most widely used, reliable method for assessing cerebral vasoreactivity and autoregulatory function. Using TCD changes in cerebral blood flow velocity (BFV) in proximal intracranial arteries can be measured with excellent temporal sensitivity [64–66]. Several TCD methods have been developed to assess cerebrovascular function (e.g., hyperventilation test, apnea test and acetazolamide test, thigh cuff test). In the present study, we used the common carotid artery compression (CCC) test, which is a validated method to estimate cerebral vasoreactivity and autoregulatory function [67–71]. Importantly, the CCC test is the only functional TCD test that mimics the hemodynamic conditions associated with clamping during carotid reconstruction surgery [72]. During CCC, the perfusion pressure in the ipsilateral middle cerebral artery (MCA) is significantly reduced, which is associated with an immediate decrease in BFV. When compression is released, a rapid transient increase in BFV is measured. This phenomenon is known as the transient hyperemic response (THR) from which cerebral vasoreactivity can be estimated [73–75]. Due to the significant changes in blood pressure [76, 77] and BFV occurring in a short period of time, the CCC test is often interpreted primarily as a dynamic autoregulation test. Yet, in addition to the pressure-sensitive autoregulatory myogenic function of the cerebral resistance arteries and arterioles, the characteristics of the transient hyperemic response are likely influenced by local vasoregulatory mechanisms activated by the sudden decline in oxygen delivery, shear stress-induced, endothelium-mediated regulation of vascular tone and arterial wall stiffness as well. Thus, analysis of transient hyperemic responses in CAS patients may provide useful information on multiple aspects of macro- and microvascular health. We performed simultaneous bilateral measurement of BFV in the right and left MCAs during unilateral CCC to characterize compensatory inter-hemispheric blood flow as well.

## Materials and methods

This study was conducted in accordance with the ethical principles outlined by the Regional and Institutional Committee of Science and Research Ethics of Semmelweis University (SE-RKEB, protocol number: 256/2018) and was registered at ClinicalTrials.gov (Identifier: NCT03840265). We consecutively enrolled patients who were admitted to the Semmelweis University Department of Vascular Surgery for the evaluation of internal carotid artery (ICA) stenosis between January 2019 and September 2021. Control participants with an absence of CAS diagnosis and other major diseases were recruited by flyers distributed at the clinic and by word of mouth.

Written informed consent was obtained from all participants. The severity of ICA stenosis and circle of Willis (CoW) morphology were assessed by CT angiography in CAS patients, and patients with significant ICA stenosis (>70%) were included in the study. Severity of the ICA stenosis was given based on the North American Symptomatic Carotid Endarterectomy Trial (NASCET [78]) criteria [79]. Transcranial Doppler (TCD) tests were performed only if an appropriate temporal insonation bone window was present, and exclusion criteria included features such as atrial fibrillation, extensive emboligenic plaques in the common carotid artery, and carotid sinus hyperesthesia that may compromise the performance or evaluation of the functional TCD tests. Symptomatic CAS was defined when an ipsilateral non-lacunar ischemic event was present (TIA or minor stroke in the previous 6 months), while patients with asymptomatic CAS had none of the aforementioned factors present. We collected patients’ demographic and anthropometric data (sex, age, BMI), medical history (hypertension, diabetes mellitus, smoking status, ischemic heart disease, chronic lung disease, contralateral stenosis), and results of standard laboratory tests (including thrombocyte level, serum cholesterol, low-density lipoprotein cholesterol [LDL], high-density lipoprotein cholesterol [HDL], triglyceride level, creatinine, estimated glomerular filtration rate [eGFR]). Previous medical history was based on patient interviews and prior medical documentation.

### Study protocol: transcranial Doppler sonography

The Doppler Laboratory of the Department of Neurology at Semmelweis University performed TCD examinations on patients. A bilateral, fixed TCD transducer (2 MHz, DWL Multi-Dop T2, Sipplingen, Germany) was used to record blood flow velocity in the MCAs while the patient was in a supine position and at rest through the transtemporal insonation window at a depth of 50–55 mm [80, 81]. The transducers were fixed in a head frame and adjusted for the maximum signal strength. The analog output of the TCD equipment was the envelope that was derived from the maximum of the flow velocity power spectrum following fast-Fourier transformation. Continuous, non-invasive, beat-to-beat arterial blood pressure monitoring was performed using radial artery applanation tonometry (Colin-BP508, Hayashi Komaki Aichi, Japan), which was calibrated with a standard upper arm arterial blood pressure cuff on the contralateral side. Heart rate was measured with a 3-lead surface ECG, and respiratory rate was monitored using exhaled air CO2 (EtCO2) with a capnograph connected to a nasal cannula (Colin-BP508, Hayashi Komaki Aichi, Japan).

**Fig. 1:**
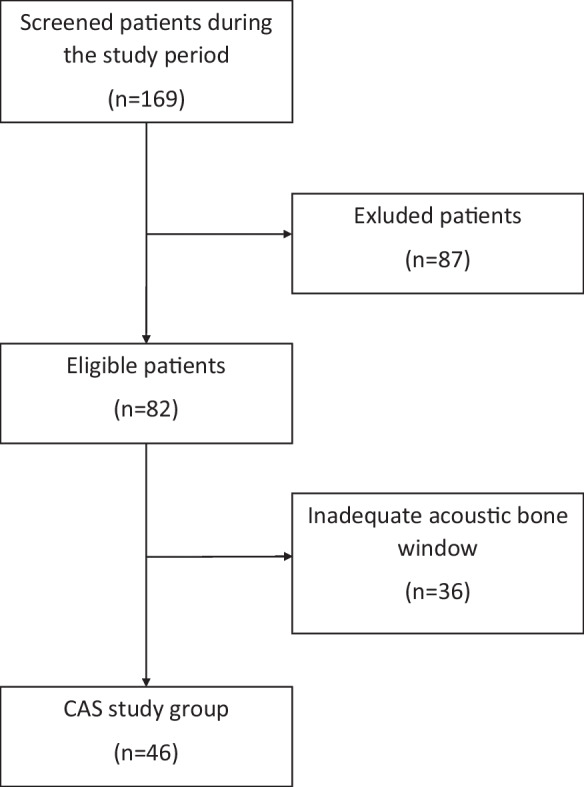
Study group flow chart

A baseline (BL) registration was recorded after a minimum rest period of 15 min. Radial arterial blood pressure was calibrated before each test, and patients were monitored clinically during the tests constantly. If there were any complaints, the tests were suspended. Of the 82 patients who met the inclusion criteria, those who were unable to have TCD measurements due to a lack of an insonation window were excluded. TCD signal quality criteria were also used for patient selection. Only signals of appropriate quality that allowed for long-term monitoring were accepted, while artifact-distorted registrations with poor quality were deemed unsuitable for the study. To ensure reproducibility and achieve the best signal-to-noise ratio, the tests were performed three times. The technically and qualitatively most optimal registration was evaluated for each patient. The compression with inadequate efficiency and improper patient cooperation can lead to distortion during the CCC test. In this study, to avoid these biases, the compression was considered effective when the blood flow velocity (BFV) reduction was at least 40% [75]. A 10-s compression was used based on the results of previous studies, as this duration can achieve the maximum THR, and a longer duration of compression does not increase the THR [75]. After excluding patients with sub-optimal measurements, measurements of 5 control participants and 46 CAS patients were found eligible for further analysis (Fig. 1).

### Data processing

The analog signals were digitized simultaneously on 5 channels (TCD1, TCD2, ECG, capnograph, tonometry) at a sampling frequency of 500 Hz. Raw data for each patient were saved in European Data Format (EDF) files, which were imported into LabChart software (AdInstruments, LabChart ver. 8, Colorado Springs, CO, USA) for digital filtering and segmentation. The resulting pre-processed data segments were stored in separate text files, which were then processed by an in-house Python code. The code linearly interpolated the five channels into 0.5-s intervals and exported the interpolated data to Microsoft Excel. The amplitude and time variables of the cerebrovascular reactivity were subsequently calculated.

### Calculation of BFV variables

**Fig. 2:**
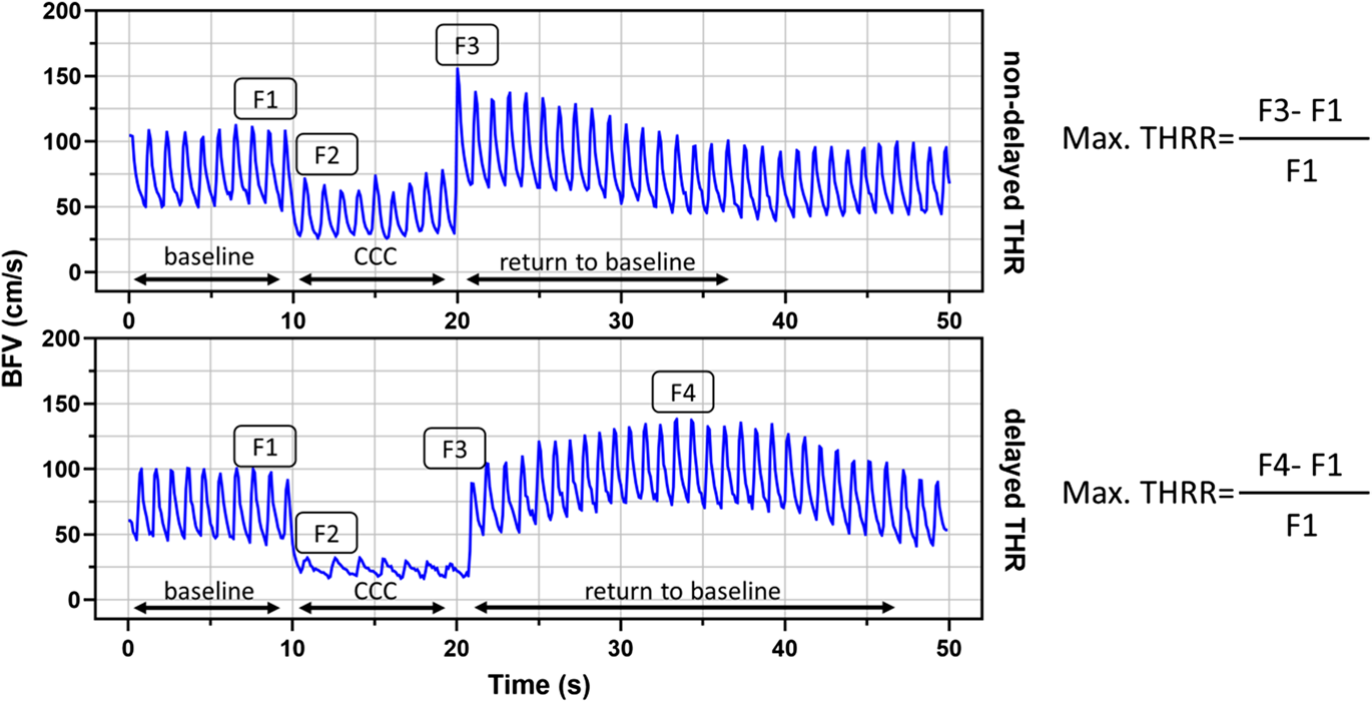
Representative registration of patients with carotid artery stenosis (CAS), illustrating non-delayed transient hyperemic response (THR) in the upper panel and delayed THR in the lower panel. Equations for calculating the maximal transient hyperemic response ratio (THRR) are provided for each group. BFV denotes blood flow velocity, CCC represents the common carotid artery compression test, RTB indicates return to baseline, F1 represents baseline BFV, F2 represents initial BFV during CCC, F3 corresponds to the early peak BFV, and F4 denotes the maximal BFV if the maximum blood flow velocity did not occur immediately after the end of CCC, but after a few cardiac cycles

**Fig. 3:**
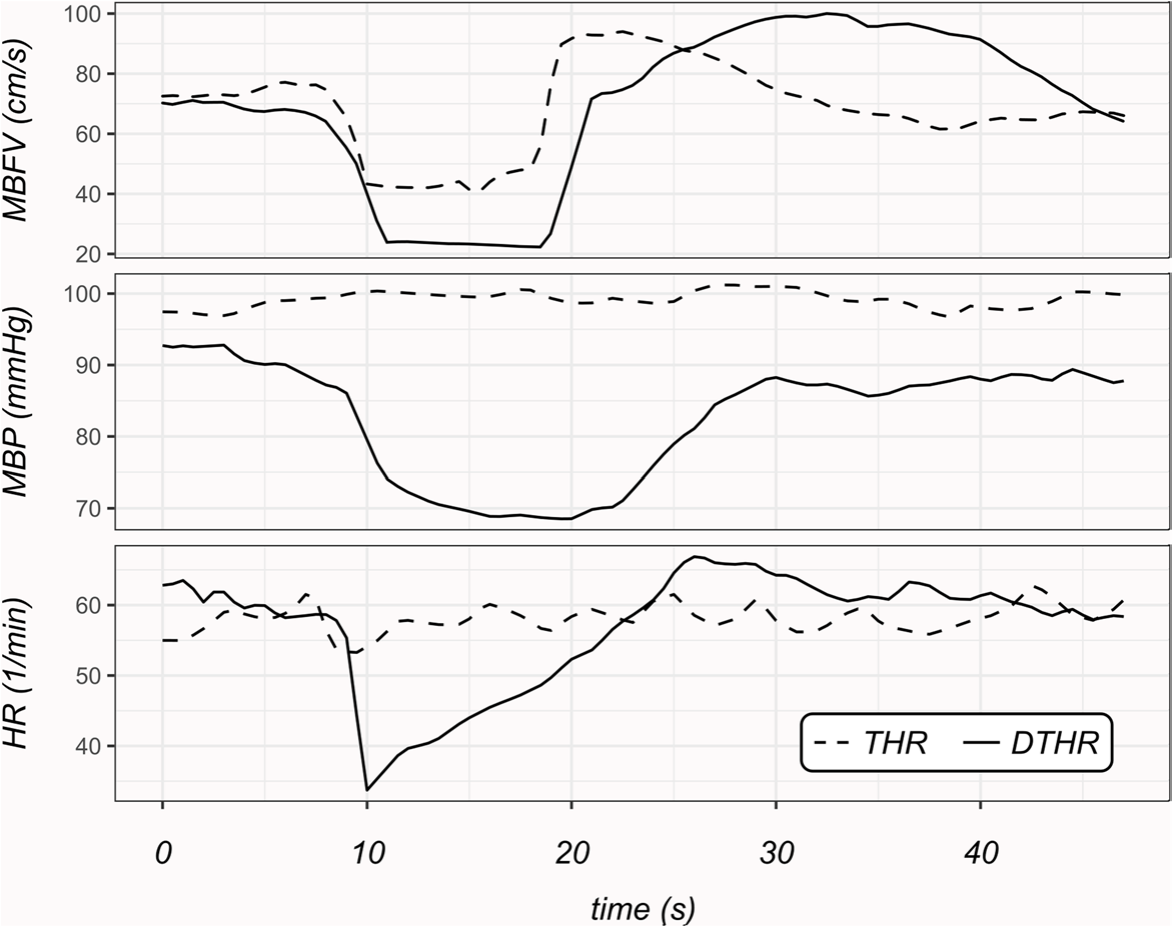
Representative recordings of patients with carotid artery stenosis (CAS) categorized into two groups based on the dynamics of transient hyperemic response: normal (THR) and delayed-type transient hyperemic response (DTHR). The recordings include mean blood flow velocity (MBFV), mean blood pressure (MBP), and heart rate (HR) measurements

The response to CCC stimulus can be described as occurring in two distinct phases, each characterized by changes in opposite directions (Figs. 2 and 3). Initially, the dilation of downstream resistance vessels induced by the pressure drop leads to an increase in blood flow (and thereby flow velocity) in the MCA after the compression is released. This increase often surpasses the baseline value observed under normal conditions which is commonly referred to as the THR. This hyperperfusion state triggers an opposite process, namely, pressure-induced (myogenic) vasoconstriction in the resistance arteries and arterioles. The return of cerebral blood flow velocity in the MCA to resting levels following this process is likely due to a combination of active myogenic [43, 82] and shear stress-induced [57, 83] vasoconstriction.

The BFV variables were calculated based on the distinctive changes observed in the MCAs during the CCC test. The analysis identified four distinct phases and peaks (F1–F4) (see Fig. 2). The baseline value (F1) was recorded immediately prior to the CCC test. F2 was defined as the BFV plateau that occurred immediately after the start of the CCC. F3 referred to the early peak of BFV increases that occurred immediately after the end of the CCC. This reaction type was considered a normal transient hyperemic response. In some patients, the maximum BFV response was not observed immediately after the end of CCC, but rather after a few cardiac cycles (F4). This type of BFV response was defined as a delayed transient hyperemic response.

The transient hyperemic response ratio (THRR) was calculated as the increase in BFV measured immediately after CCC, expressed relative to baseline ((F3 − F1)/F1). THRR was determined using previously published methods [75, 84, 85] based on mean BFV. Delayed THRR was calculated as the maximal BFV increase after the release of CCC, expressed relative to baseline ((F4 − F1)/F1). Patients could be categorized into intact vasoreactivity (THRR > 10%) and impaired vasoreactivity (THRR < 10%) groups, based on the literature suggesting that a minimum BFV increase of 10% indicates intact cerebral vasoreactivity [75].

To estimate compressible perfusion, the percentage by which BFV decreases during CCC compared to baseline was calculated.

Cumulative blood flow and repayment during post-CCC hyperemia were estimated by calculating the area under the curve (AUC) from the mean BFV curve, from the end of CCC to the return to baseline. Excess cumulative blood flow was estimated as the integrated BFV during THR minus the integrated pre-CCC BFV for a period corresponding to the duration of the hyperemic response.

Cerebrovascular resistance (CVR) was defined as the quotient of mean arterial blood pressure and BFV [86]. CVR was determined at F1, F3, and F4 points. CVR-THRR and CVR-delayed THRR values were defined similarly to THRR, where the change in resistance from baseline (CVR_F1_) to the minimum CVR value after compression (F3 or F4) was calculated as CVR_THRR_ or CVR_delayed_THRR_ (CVR_THRR_ = (CVR_F3_ − CVR_F1_)/CVR_F1_ and CVR_delayed_THRR_ = (CVR_F4_ − CVR_F1_)/CVR_F1_). CR_THRR_ was calculated for all patients, while CR_delayed_THR_ values were calculated for patients with delayed THR reaction.

Return to baseline time (RTB) was defined as the time required for BFV to return to baseline value after manual compression release (Fig. 2). The decrease in arterial blood pressure during compression was considered if a change larger than 2 standard deviations in arterial blood pressure was present compared to baseline measurements. Heart rate reduction was considered based on a similar calculation. Time to peak (TTP) was defined as the time (in seconds) required for maximum BFV increase (F3 or F4) to occur from the end of CCC.

### Assessment of CoW morphology

Two expert neuroradiologists evaluated CoW morphology using CT angiography. Segments were classified as normal (diameter > 0.8 mm), hypoplastic (diameter ˂ 0.8 mm), or aplastic (non-visualized) based on previous studies [61, 87]. A compromised CoW was defined as at least one of the posterior or anterior CoW segments (anterior communicating artery, anterior cerebral artery A1 segment, posterior communicating artery) being aplastic or hypoplastic. All segments were present in the intact CoW group.

### Statistical analysis

The statistical analysis and visualization of data was performed with GraphPad Prism 9.2.0 program. The significance level was defined as *p* < 0.05 for all tests. Categorical variables of groups were compared with *χ*^2^ test or Fisher’s exact test. Normal distribution of data was determined using the Kolmogorov-Smirnov test. Outlier values were excluded using the ROUT test (*Q* = 1%), and the number of excluded outliers are reported where applicable. For multigroup comparisons of normally distributed data, the one-way ANOVA test with post hoc Tukey’s test or two-way ANOVA with post hoc Bonferroni test was conducted. For comparisons of non-normally distributed data, Mann-Whitney *U* test and Kruskal-Wallis test with Dunn’s post hoc test were used. The relationship between non-normally distributed data was investigated using the Spearman correlation test.

## Results

Our study included a total of 46 patients, comprising 14 women (mean age: 69 ± 7.4 years) and 32 men (mean age: 68 ± 6.8 years), as shown in Fig. 1. Almost one-third of the patients reported being smokers, and one-third had diabetes mellitus. All patients had treated hypertension. Of the total patients, 15 (32%) had symptomatic ICA stenosis, while 31 (67%) had asymptomatic ICA stenosis. None of the patients experienced any discomfort or adverse reaction during the CCC tests.

### Comparison of patients with asymptomatic vs. symptomatic CAS

Table 1 presents the baseline characteristics of patients with asymptomatic and symptomatic CAS. Notably, compromised CoW was found to be significantly more prevalent in the symptomatic group, affecting 40% of patients as compared to only 10% of asymptomatic patients. Additionally, the symptomatic CAS group showed a lower incidence of delayed THR, with only 27% of patients exhibiting this symptom, as opposed to 58% in the asymptomatic CAS group (*p* = 0.046).

**Table 1:** Demographic and clinical characteristics of patients with carotid artery stenosis (CAS) grouped by symptomatic status

	Symptomatic (*n* = 15)	Asymptomatic (*n* = 31)	*p* value	Statistical test
Age	67 ± 1.4	68 ± 1.3	0.68	*t*-Test
Sex (female/male) [*n*, (%)]	4/11 (27%/73%)	10/21 (32%/68%)	0.7	*χ*^2^ test
BMI (median [IQR])	25.6 [23.1 to 27.1]	28.7 [27.1 to 30.8]	0.16	Mann-Whitney *U* test
Hypertension [*n*, (%)]	13 (87%)	30 (94%)	0.19	*χ*^2^ test
Diabetes [*n*, (%)]	6 (40%)	12 (39%)	0.93	*χ*^2^ test
Smoking [*n*, (%)]	8 (53%)	8 (39%)	0.067	*χ*^2^ test
Degree of stenosis (mean %)	80%	80%	0.26	Mann-Whitney *U* test
Contralateral stenosis [*n*, (%)]	2 (13%)	7 (23%)	0.46	*χ*^2^ test
Compromised circle of Willis [*n*, (%)]	6 (40%)	9 (10%)	0.015*	*χ*^2^ test
Delayed THR peak present [*n*, %]	4 (27%)	18 (58%)	0.0625	Fisher’s exact test
Impaired vasoreactivity [*n*, %]	6 (40%)	11 (35%)	0.76	*χ*^2^ test

Data are presented as median [interquartile range] for continuous variables and as number of patients and percentage for categorical variables. *BMI*, body mass index; *THR*, transient hyperemic response; *IQR*, interquartile range; *SD*, standard deviation. The table includes the following parameters: age, sex distribution, BMI, presence of hypertension, diabetes, and smoking, degree of stenosis, contralateral stenosis, compromised circle of Willis, and delayed THR peak present. The symptomatic status of the patients (i.e., symptomatic or asymptomatic) is used as the grouping variable, * = significant.

**Fig. 4:**
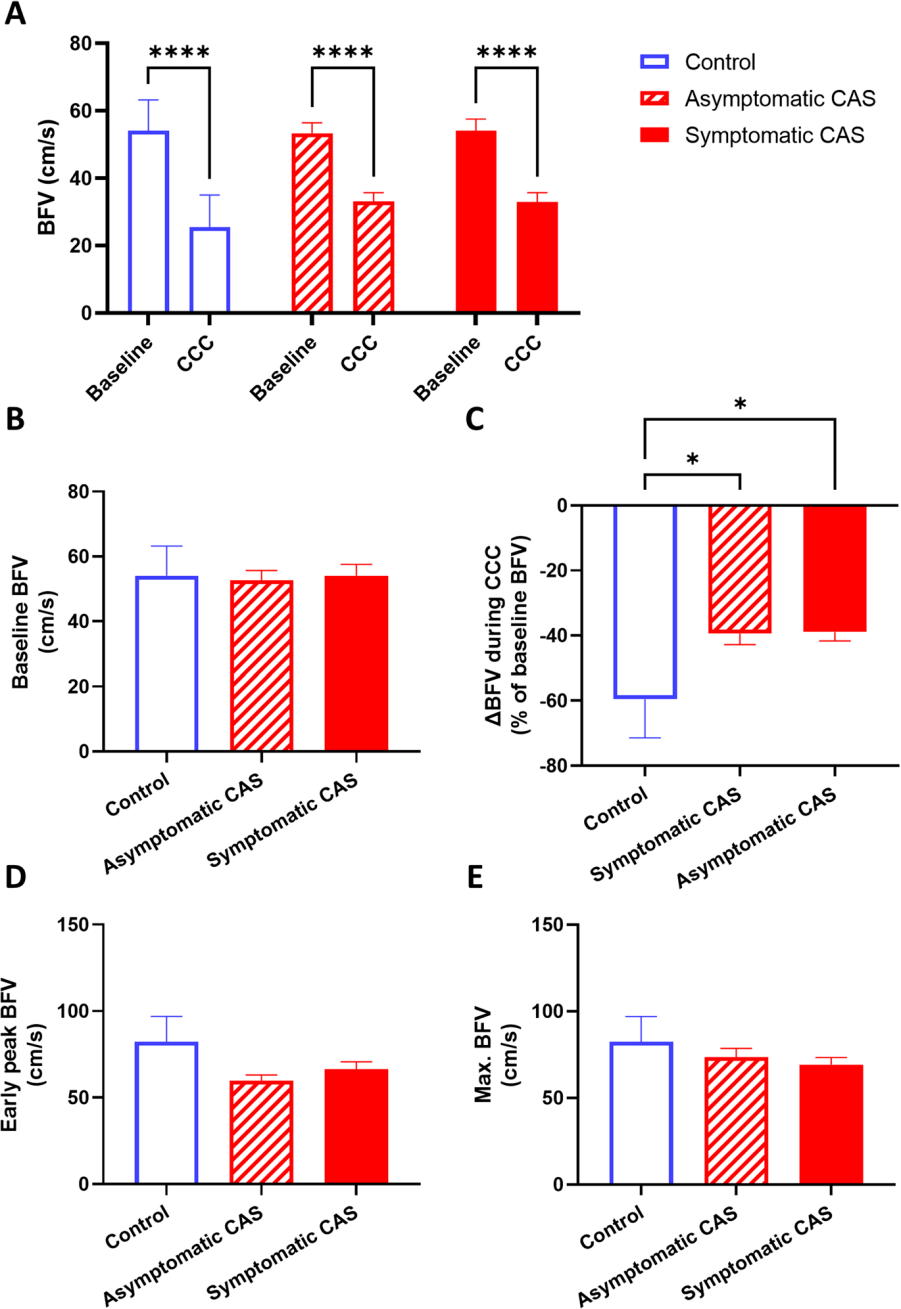
Middle cerebral artery (MCA) blood flow velocity (BFV) measured by transcranial Doppler sonography in control, asymptomatic CAS, and symptomatic CAS groups under resting and common carotid artery compression (CCC) testing conditions. **A** Comparison of baseline BFV and BFV during CCC. Two-way ANOVA with post hoc Bonferroni test was performed, and the results of pairwise comparisons are displayed. **B** Comparison of baseline BFV among the groups. **C** Comparison of the maximal change in BFV during CCC. **D** Comparison of the early peak BFV after CCC release. **E** Comparison of the maximal BFV during the hyperemic response. For **B**–**E**, one-way ANOVA was conducted on normally distributed data, followed by post hoc Tukey’s test. The results of pairwise comparisons are presented. The mean values with standard error of the mean (SEM) are depicted. Significance levels are indicated as follows: *p < 0.05, **p < 0.01, ***p < 0.001, ****p < 0.0001

Blood flow velocity (BFV) was compared in the middle cerebral artery (MCA) in three groups—asymptomatic CAS patients, symptomatic CAS patients, and controls—before, during, and after CCC was performed, as shown in Fig. 4. Two-way ANOVA testing revealed a significant effect of CCC, causing a significant change in BFV (*F*[1, 47] = 200.2, *p* < 0.01), and post hoc testing confirmed the significant decrease of BFV in all three groups (Fig. 4A). However, baseline BFV did not differ significantly between the groups (Fig. 4A–B). While there was a significant effect of patient group in the change of BFV caused by CCC (*F*[2, 46] = 3.843, *p* = 0.03), post hoc testing only found differences between the control and CAS groups (control vs. asymptomatic CAS: *p* = 0.02; control vs. symptomatic CAS: *p* = 0.04), but not between the two CAS groups. No differences were found in the early peak (F3) or maximal (F3 or F4) BFV in the three groups.

**Fig. 5:**
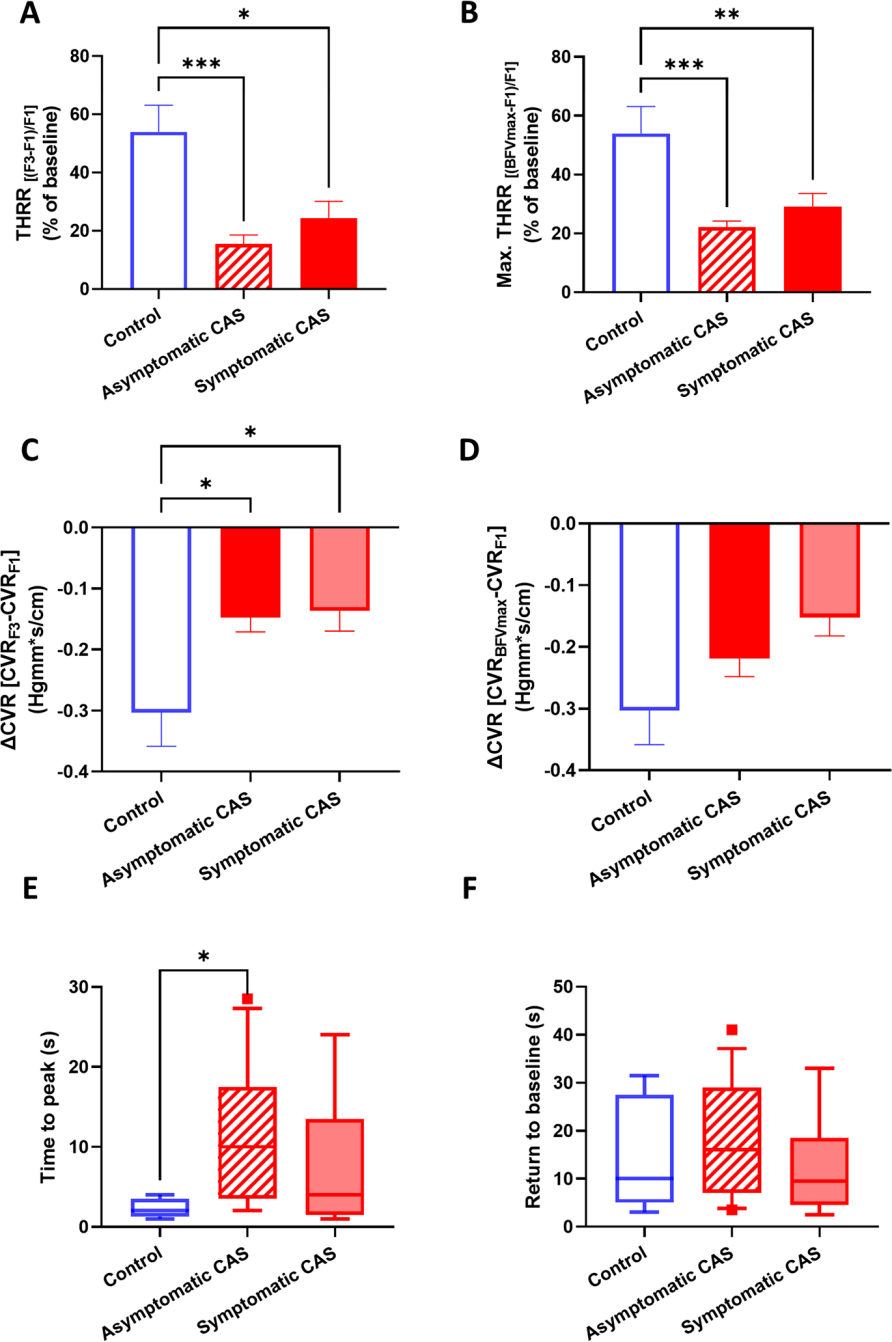
Parameters describing the transient hyperemic response (THR) elicited by the common carotid artery compression (CCC) test in control subjects, asymptomatic CAS patients, and symptomatic CAS patients. **A** Transient hyperemic response ratio (THRR) calculated from the first peak of recorded blood flow velocity (BFV) following CCC release. **B** Transient hyperemic response ratio (THRR) calculated from the maximal BFV recorded after CCC release. **C** Change in cerebrovascular resistance index (CVR) at the first peak of BFV after CCC release. **D** Change in cerebrovascular resistance index (CVR) at the maximal BFV after CCC release. **E** Time elapsed to reach the maximal BFV after CCC release. **F** Time for BFV to return to baseline after CCC release. For **A**–**D**, one-way ANOVA was conducted on normally distributed data, followed by post hoc Tukey’s test for pairwise comparisons. The mean values with standard error of the mean (SEM) are depicted. For **E**–**F**, the Kruskal-Wallis test was performed due to non-normally distributed data, and post hoc Dunn’s test was used for pairwise comparisons. The median values with interquartile range (boxes) and 5–95 percentiles (whiskers) are displayed. Significance levels are indicated as follows: *p < 0.05, **p < 0.01, ***p < 0.001, ****p < 0.0001

Transient hyperemic responses evoked by CCC were compared in the same three groups. Significant differences in THRR were found between the groups (*F*[2, 46] = 9.16, *p* < 0.01), and post hoc testing revealed differences between controls and both asymptomatic CAS (*p* < 0.01) and symptomatic CAS (*p* < 0.01), but not between the two CAS groups (Fig. 5A). When maximal THRR was evaluated, ROUT test identified five outliers in the asymptomatic CAS group, who were excluded. Similarly to THRR, one-way ANOVA found a significant effect of patient group on maximal THRR (*F*[2, 40] = 10.57, *p* = 0.03), and pairwise comparisons revealed differences between the control and CAS patient groups (asymptomatic CAS: *p* < 0.01; symptomatic CAS: *p* < 0.01, Fig. 5B).

When the change in cerebrovascular resistance (ΔCVR) was compared, significant group effects were found (*F*[2, 46] = 3.396, *p* = 0.04), and post hoc test highlighted differences between the control and CAS groups only (asymptomatic CAS: *p* = 0.04, symptomatic CAS: *p* = 0.04, Fig. 5C). No significant differences were found when the same analysis was performed on ΔCVR calculated from CVR at BFVmax (Fig. 5D). Significant differences were found in the timing required to reach BFVmax between the three groups (*p* < 0.01), as revealed by Kruskal-Wallis test, and post hoc testing found significant differences between the controls and asymptomatic CAS patients (*p* = 0.01, Fig. 5E). However, return to baseline (RTB) did not show significant differences when compared among the three groups (Fig. 5F).

**Fig. 6:**
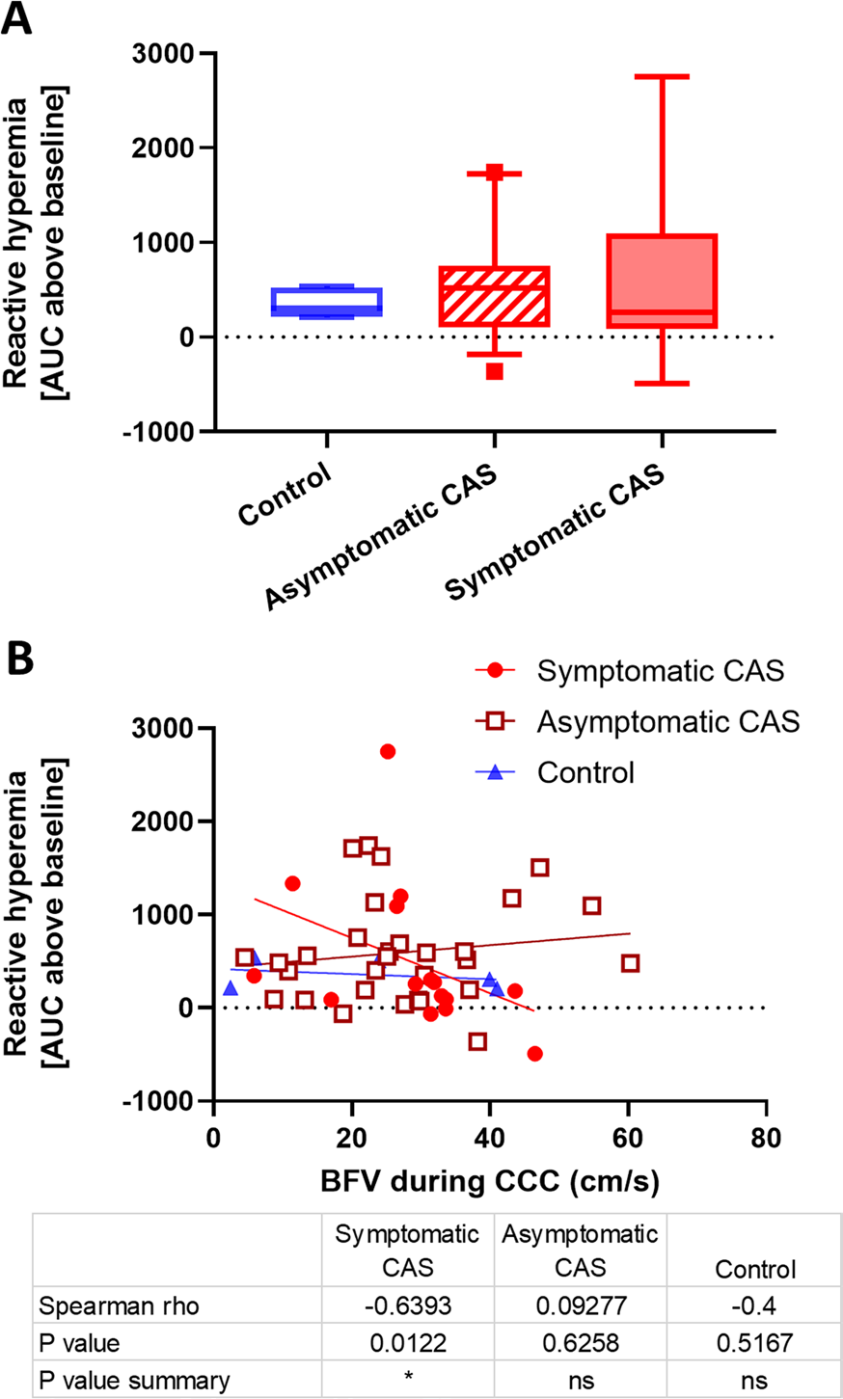
Reactive hyperemia responses in control subjects, asymptomatic CAS patients, and symptomatic CAS patients induced by common carotid artery compression (CCC) testing. **A** Area under the curve (AUC) of hyperemic responses following CCC. The Kruskal-Wallis test was performed due to non-normally distributed data, and post hoc Dunn’s test was applied. No significant differences were found in the post hoc testing. The median values with interquartile range (boxes) and 5–95 percentiles (whiskers) are shown. **B** Correlation analysis of the evoked reactive hyperemia responses plotted against the blood flow velocity (BFV) measured during CCC. Linear regression lines are fitted to display linear trendlines, and the Spearman’s rho and *p* values are provided in the accompanying table

The CCC-evoked reactive hyperemia was measured as the area under the curve (AUC) above the baseline, and no differences were found when reactive hyperemia of the three groups was compared (Fig. 6A). However, when the correlation between the reactive hyperemia AUC and BFV during CCC that evoked the reactive hyperemia was analyzed, significant correlation was found only in the symptomatic CAS group (Fig. 6B).

### Comparison of CAS patients with non-delayed vs. delayed transient hyperemic responses

The present study also aimed to compare patients with CAS who had non-delayed vs. delayed transient hyperemic responses following CCC. Table 2 presents a comparison of the characteristics between CAS patients with delayed and non-delayed THRs. Of the total 46 CAS patients included in the study, 22 (48%) exhibited delayed THR while 24 (52%) showed non-delayed THR. The delayed THR group was found to have a significantly higher mean age compared to the non-delayed THR group.

**Table 2:** Comparing characteristics of non-delayed THR and delayed THR groups

Data	Non-delayed THR group	Delayed THR group	Test	*p* value
Demographic data				
Number of patients (*N*/%)	24/52	22/48	NA	
Age, years median (IQR)	67 (62.3–72.8)	70 (65.5–72.3)	Mann-Whitney *U*	0.46
Sex, male (*N*/%)	15/65	14/52	Chi-square	0.36
Medical history				
Hypertension (*N*/%)	24/100	21/100	NA	
Symptomatic (*N*/%)	11/45	4/18	Fisher’s exact	0.06
Contralateral sign. stenosis or occlusion (*N*/%)	3/13	7/30	Fisher’s exact	0.14
Diabetes (*N*/%)	9/39	7/30	Chi-square	0.53
Smoking (*N*/%)	7/31	7/30	Fisher’s exact	0.92
Ischemic heart disease (*N*/%)	6/26	9/39	Chi-square	0.77
Chronic lung disease (*N*/%)	2/8	2/8	Fisher’s exact	0.69
Multimodal TCD metrics				
THRR (median, IQR)	***0.28 (0.2–0.3)***	***0.01 (−0.03–0.1)***	Mann-Whitney *U*	***˂0.001***
RTB (median, IQR)	***6.9 (4.4–10.0)***	***27.8 (15.9–29.4)***	Mann-Whitney *U*	***˂0.001***
Baseline CVR (median, IQR)	***−0.17 (−0.4–0.09)***	***−0.09 (−0.3–0.1)***	Mann-Whitney *U*	***0.04***
HR decrease (*N*/%)	18/75	19/86	Fisher’s exact	0.27
ABP decrease (*N*/%)	12/52	19/82	Fisher’s exact	0.02
Impaired vasoreactivity (*N*/%)	3/13	8/36	Fisher’s exact	***˂0.001***

Data are presented as median [interquartile range] for continuous variables and as number of patients and percentage for categorical variables. *THR*, transient hyperemic response; *IQR*, interquartile range;  The table includes the following parameters: age, sex distribution, presence of hypertension, diabetes, and smoking, symptomatic senosis, contralateral stenosis or occlusion, ischemic heart disease, chronic lung disease, *THRR*, transient hyperemic response ratio; *RTB*, return to baseline; baseline *CVR*, cerebrovascular resistance; *HR*, heart rate; *ABP*, arterial blood pressure. The non-delayed THR and delayed THR status of the patients is used as the grouping variable. Significant difference was found by THRR, RTB, baseline CVR, HR decrease, *ABP* decrease and impaired vasoreactivity

**Fig. 7:**
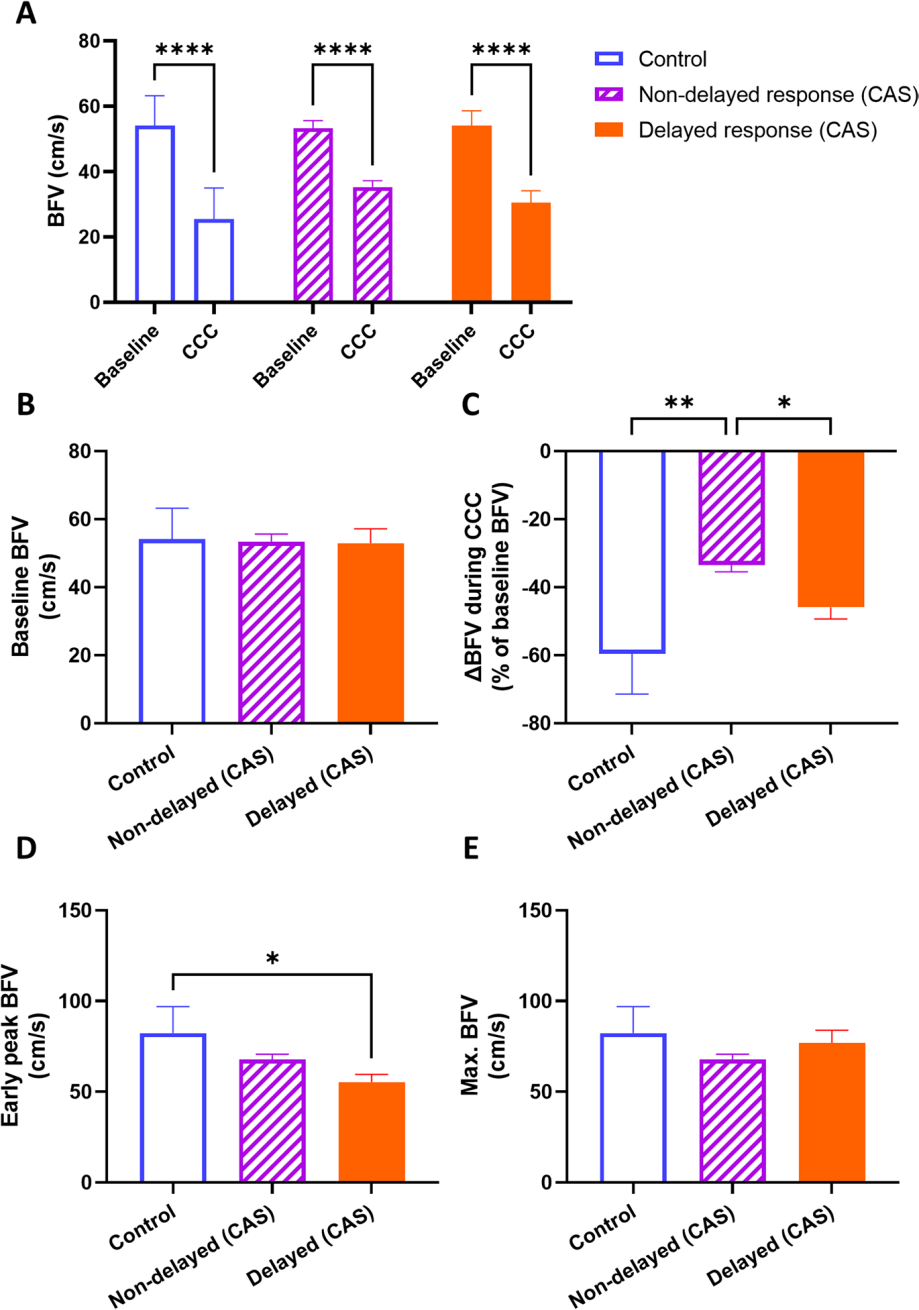
Middle cerebral artery (MCA) blood flow velocity (BFV) measured by transcranial Doppler sonography in different carotid artery stenosis (CAS) patient groups classified by the presence of delayed or non-delayed transient hyperemic response during resting and common carotid artery compression (CCC) testing conditions. **A** Comparison of baseline BFV and BFV during CCC. Two-way ANOVA with post hoc Bonferroni test was conducted, and the results of pairwise comparisons are displayed. **B** Comparison of baseline BFV among the patient groups. **C** Comparison of the maximal change in BFV during CCC. **D** Comparison of the early peak BFV after CCC release. **E** Comparison of the maximal BFV during the hyperemic response. For **B**–**E**, one-way ANOVA was performed on normally distributed data, followed by post hoc Tukey’s test for pairwise comparisons. The mean values with standard error of the mean (SEM) are depicted. Significance levels are indicated as follows: *p < 0.05, **p < 0.01, ***p < 0.001, ****p < 0.0001

We conducted a comparison of BFV and CCC-evoked transient hyperemic response parameters in CAS patients with non-delayed THR, delayed THR, and controls. Baseline BFV did not differ significantly between the groups (Fig. 7A–B). Two-way ANOVA revealed a significant effect of CCC intervention on BFV (*F*[1, 47] = 222.1, *p* < 0.01), with a significant decrease in all three groups (*p* < 0.01, Fig. 7A). The change of BFV caused by CCC was significantly affected by the patient group (*F*[2, 46] = 8.532, *p* < 0.01), and post hoc testing found significant differences between the control vs. non-delayed THR (*p* < 0.01) and non-delayed THR CAS vs. delayed THR CAS (*p* = 0.02) comparisons (Fig. 7C). The early peak (F3) BFV was also influenced by the patient group (*F*[2, 46] = 5.125, *p* < 0.01), with a pairwise comparison-detected significant difference between the control and delayed THR CAS group (*p* = 0.02, Fig. 7D). However, no differences were found in the maximal (F3 or F4) BFV in the observed three groups (Fig. 7E).

**Fig. 8:**
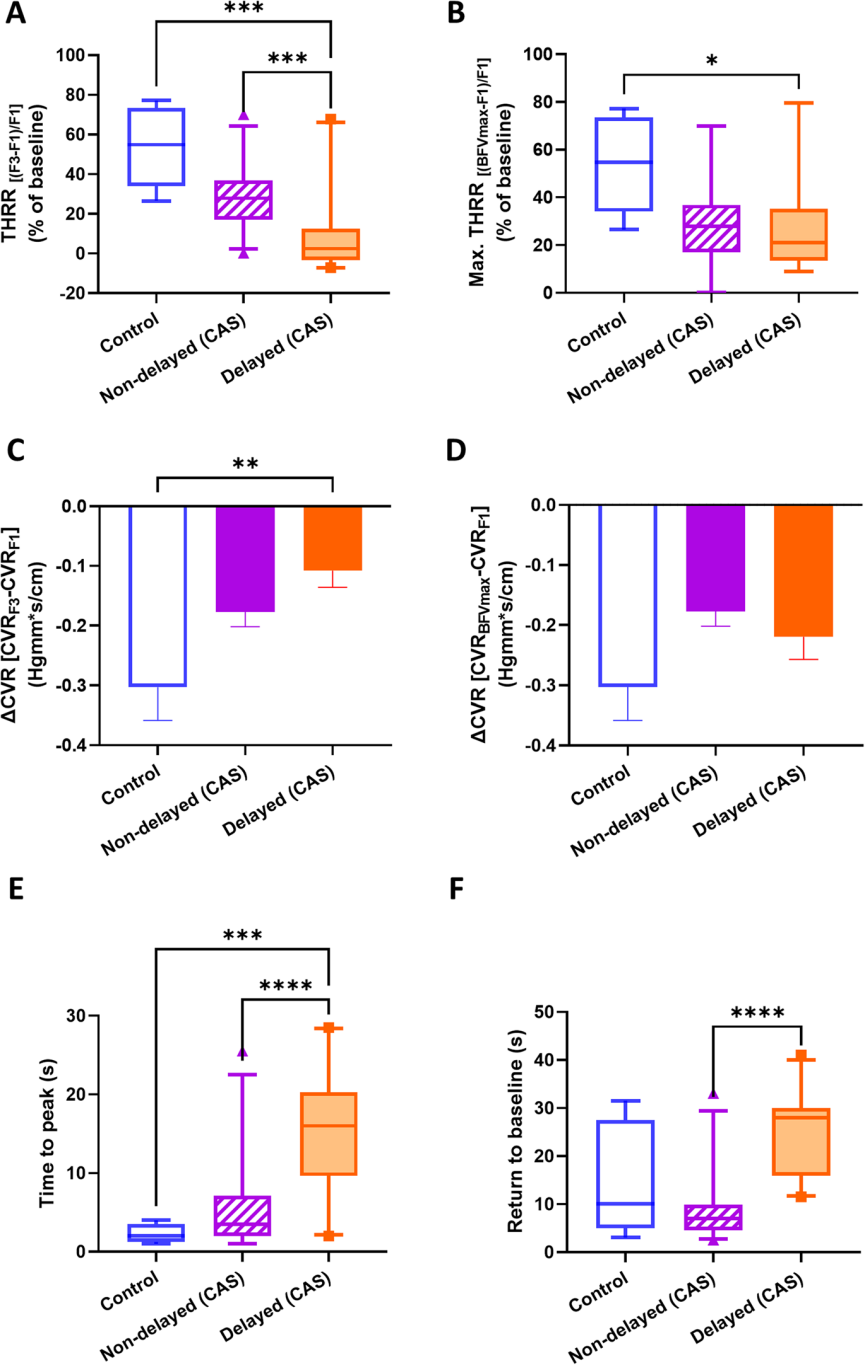
Parameters characterizing the transient hyperemic response (THR) induced by the common carotid artery compression (CCC) test in control subjects and carotid artery stenosis (CAS) patients with delayed and non-delayed transient hyperemic response. **A** Transient hyperemic response ratio (THRR) calculated from the first peak of blood flow velocity (BFV) recorded after CCC release. **B** Transient hyperemic response ratio (THRR) calculated from the maximal BFV recorded after CCC release. **C** Change in cerebrovascular resistance index (CVR) at the first peak of BFV after CCC release. **D** Change in cerebrovascular resistance index (CVR) at the maximal BFV after CCC release. **E** Time elapsed to reach the maximal BFV after the release of CCC. **F** Time taken for BFV to return to baseline after CCC release. The Kruskal-Wallis test was used to analyze non-normally distributed data for **A**–**B**, **E**–**F**, and post hoc Dunn’s test was applied for pairwise comparisons. The results are presented as median values with interquartile range (boxes) and 5–95 percentiles (whiskers). One-way ANOVA with post hoc Tukey’s test was conducted on normally distributed data for **C**–**D**, and the pairwise comparisons are shown as mean ± SEM. Statistical significance is indicated as follows: * for *p* < 0.05, ** for *p* < 0.01, *** for *p* < 0.001, and **** for *p* < 0.0001

**Fig. 9:**
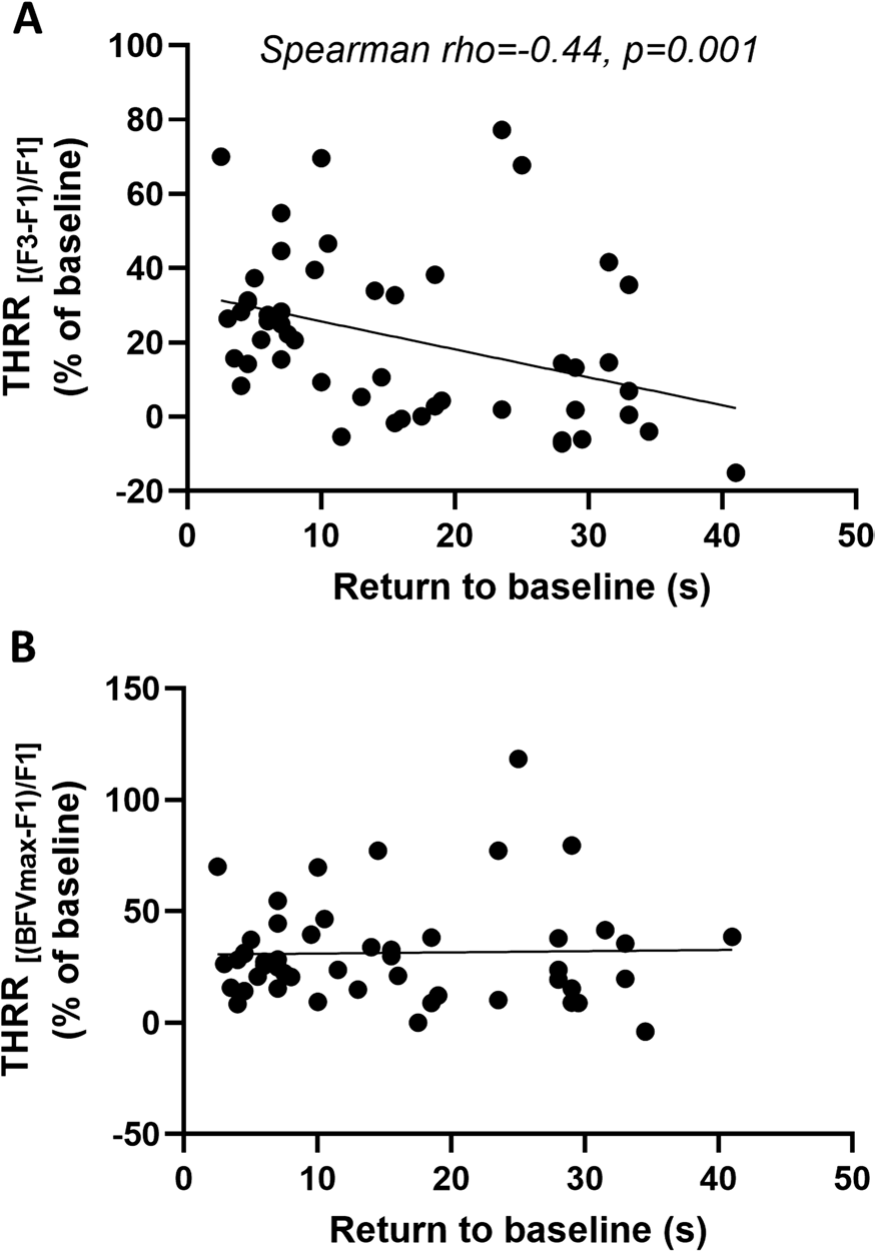
Correlation analysis of the transient hyperemic response ratios (THRR) elicited by the common carotid artery compression (CCC) test plotted against the time taken for the middle cerebral artery (MCA) blood flow velocity (BFV) to return to its baseline value. **A** THRR calculated from the first peak of BFV (F3) after CCC release. **B** THRR calculated from the maximal peak of BFV (F3 or F4) after CCC release. Spearman rho = 0.16, *p* = 0.46. Linear regression lines were fitted to display linear trends, and Spearman’s rho and *p* values are reported to indicate the strength and significance of the correlations

We also evaluated CCC-evoked transient hyperemic response parameters, including THRR (calculated at F3 waveform), maximal THRR (calculated from BFVmax), ΔCVR (calculated from CVR at F3 and BFVmax), time to peak BFV, and time for BFV to return to baseline (RTB). THRR was significantly different between groups (*p* < 0.01), and post hoc testing found differences in the controls vs. delayed THR CAS (*p* < 0.01) and non-delayed THR CAS vs. delayed THR CAS groups (*p* < 0.01, Fig. 8A). Kruskal-Wallis comparison found differences between groups in maximal THRR, and pairwise comparisons found differences between the control and delayed THR CAS group (*p* = 0.03, Fig. 8B). One-way ANOVA revealed a significant effect of patient group on ΔCVR (*F*[2, 46] = 5.338, *p* < 0.01), with post hoc tests highlighting differences between the control and delayed THR CAS groups (*p* < 0.01, Fig. 8C). However, when the same analysis was performed on ΔCVR, calculated from CVR at BFVmax, no significant differences were found (Fig. 8D). The time to peak BFV was found to be different (*p* < 0.01), and the post hoc test detected significant differences between the controls and the delayed THR CAS (*p* < 0.01) and also between the delayed THR CAS and non-delayed THR CAS (Fig. 8E). Kruskal-Wallis test also found the time for BFV to return to baseline (RTB) significantly different (*p* < 0.01), and pairwise comparisons highlighted differences between the control and the delayed THR CAS group (Fig. 8F). Additionally, we observed a significant, negative correlation between THRR (calculated with F3) and RTB values (Spearman rho = −0.44, *p* < 0.001, Fig. 9A) (Table 3), while no significant correlation was found for THRR calculated with BFV_max_ (Fig. 9B).

**Table 3:** Return to baseline time analysis

Multimodal TCD characteristics	Statistical tests for the analysis of RTB	Spearman rho	*p* value
THRR	Spearman correlation	***−0.44***	***˂0.001***
CVR-THRR	Spearman correlation	***0.21***	***0.05***
HR decrease	Mann-Whitney *U*	N/A	0.56
ABP decrease	Mann-Whitney *U*	N/A	0.31
Impaired vasoreactivity	Mann-Whitney *U*	N/A	**0.007**

*RTB*, return to baseline time; *THRR*, transient hyperemic response ratio; *CVR-THRR*, cerebrovascular resistance-transient hyperemic response ratio; *HR*, heart rate; *ABP*, arterial blood pressure. Significant correlation was found by THRR and CVR-THRR, significant difference was found by impaired vasoreactivity

### Comparison of CAS patients with intact CoW vs. CAS patients with compromised CoW

**Fig. 10:**
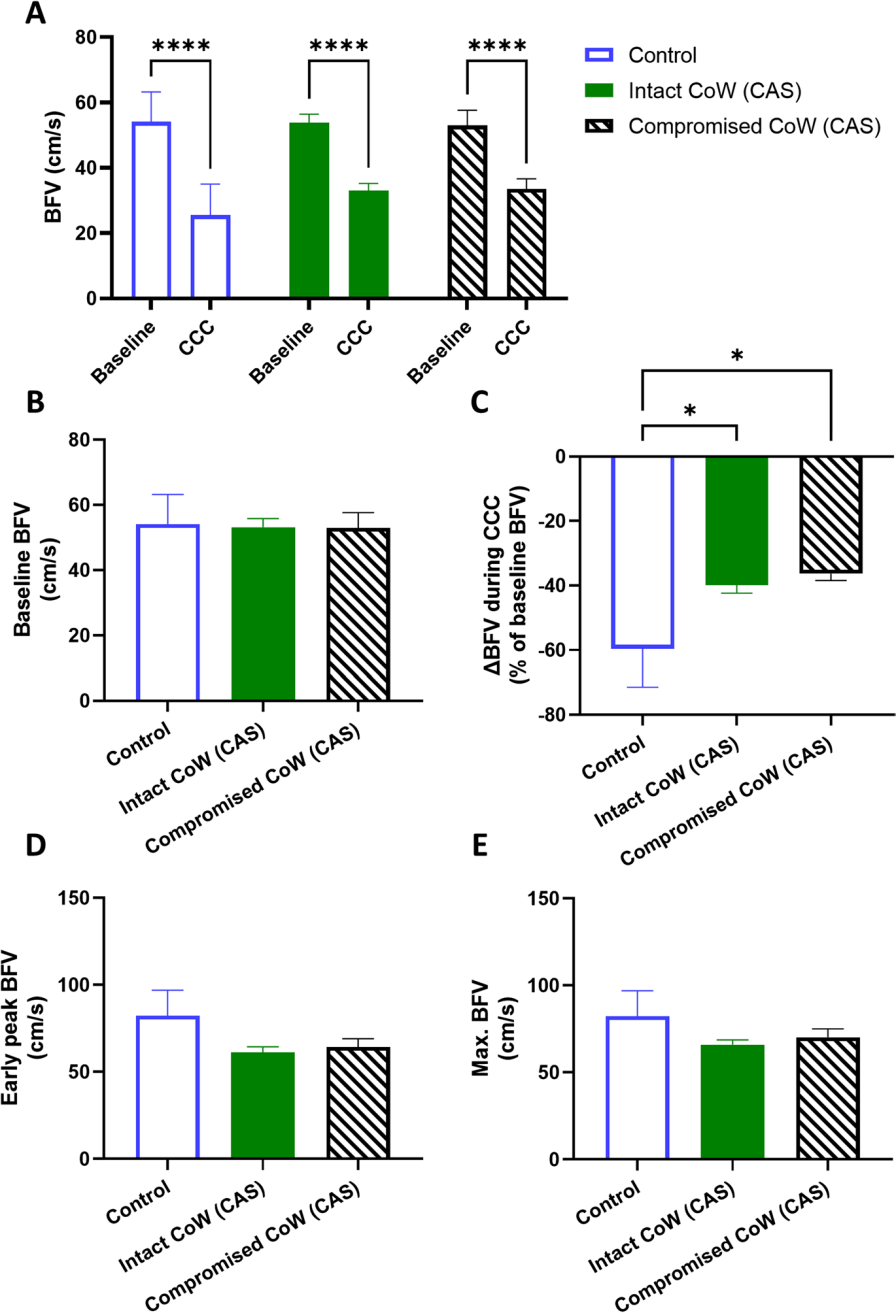
Middle cerebral artery (MCA) blood flow velocity (BFV) measured by transcranial Doppler sonography in carotid artery stenosis (CAS) patient groups categorized by the presence of an intact or compromised circle of Willis (CoW) under resting and common carotid artery compression (CCC) testing conditions. **A** Comparison of baseline BFV and BFV during CCC. Two-way ANOVA with post hoc Bonferroni test was performed, and the results of pairwise comparisons are presented. **B** Comparison of baseline BFV among the patient groups. **C** Comparison of the maximal change in BFV during CCC. **D** Comparison of the early peak BFV after CCC release. **E** Comparison of the maximal BFV during the hyperemic response. For **B**–**E**, one-way ANOVA was conducted on normally distributed data, followed by post hoc Tukey’s test for pairwise comparisons. The mean values with standard error of the mean (SEM) are depicted. Significance levels are indicated as follows: *p < 0.05, **p < 0.01, ***p < 0.001, ****p < 0.0001

**Fig. 11:**
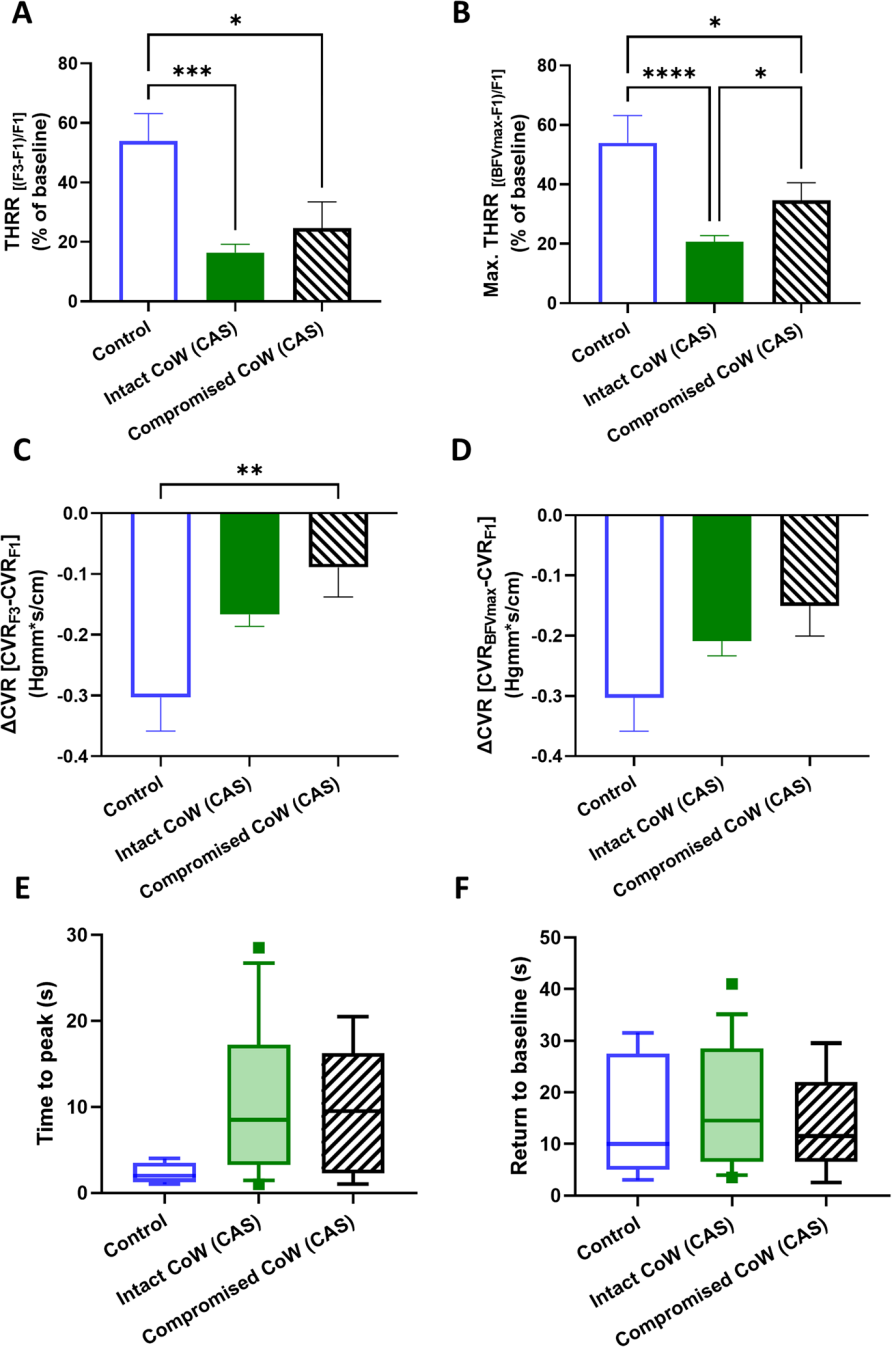
Parameters characterizing the transient hyperemic response (THR) induced by the common carotid artery compression (CCC) test in control subjects and carotid artery stenosis (CAS) patients categorized by the presence of an intact or compromised circle of Willis (CoW). **A** Transient hyperemic response ratio (THRR) calculated from the first peak of blood flow velocity (BFV) recorded after CCC release. **B** Transient hyperemic response ratio (THRR) calculated from the maximal BFV recorded after CCC release. **C** Change in cerebrovascular resistance index (CVR) at the first peak of BFV after CCC release. **D** Change in cerebrovascular resistance index (CVR) at the maximal BFV after CCC release. **E** Time elapsed to reach the maximal BFV after CCC release. **F** Time taken for BFV to return to baseline after CCC release. For **A**–**D**, one-way ANOVA was performed on normally distributed data, followed by post hoc Tukey’s test for pairwise comparisons. The mean values with standard error of the mean (SEM) are depicted. For **E**–**F**, the Kruskal-Wallis test was conducted on non-normally distributed data, followed by post hoc Dunn’s test for pairwise comparisons. The results are presented as median values with interquartile range (boxes) and 5–95 percentiles (whiskers). Significance levels are indicated as follows: *p < 0.05, **p < 0.01, ***p < 0.001, ****p < 0.0001

The study evaluated the CoW anatomy in patients with CAS and identified that 9 out of 45 patients (20%) had a compromised CoW. The subsequent analysis compared various blood flow velocity and THR parameters during CCC testing in subgroups of CAS patients with intact CoW, compromised CoW, and controls (Figs. 10 and 11).

Blood flow velocity parameters during CCC testing showed that CCC intervention significantly decreased BFV in all three groups (*p* < 0.01), without significant differences in baseline BFV (Fig. 10A–B). However, the BFV drop caused by CCC was influenced by the patient group (*F*[2, 46] = 4.134, *p* = 0.02), with significant differences between controls vs. intact CoW CAS (*p* = 0.03) and controls vs. compromised CoW CAS (*p* = 0.03) (Fig. 10C). No significant differences were found in early peak (F3) and maximal (F3 or F4) BFV among the groups (Fig. 10D–E).

Transient hyperemic responses evoked by CCC were significantly different between the control group and the CAS groups with intact (*p* < 0.01) or compromised (*p* = 0.02) CoW (Fig. 11A). The comparison of maximal transient hyperemic response calculated from BFV_max_ showed significant group effects (*F*[2, 42] = 14.11, *p* < 0.01), with significant differences found between controls vs. intact CoW CAS (*p* < 0.01), controls vs. compromised CoW CAS (*p* = 0.04), and intact CoW CAS vs. compromised CoW CAS (*p* = 0.03) (Fig. 11B).

The change in cerebrovascular resistance (ΔCVR) calculated from CVR at F3 was significantly different between patient groups (*F*[2, 46] = 4.856, *p* = 0.01), with significant differences between the control and compromised CoW CAS groups (*p* < 0.01) (Fig. 11C). However, no significant differences were observed in ΔCVR calculated from CVR at BFV_max_ (Fig. 11D) or in time to peak BFV or RTB among the three groups (Fig. 11E–F).

Overall, these findings suggest that compromised CoW in CAS patients may lead to altered cerebral hemodynamics during CCC testing, with reduced blood flow velocity and transient hyperemic response, and increased cerebrovascular resistance compared to controls and CAS patients with intact CoW.

## Discussion

The primary objective of this study was to investigate the impact of CAS on cerebral hemodynamics and autoregulation in both symptomatic and asymptomatic patients. Our results demonstrated that CAS was associated with impaired cerebral vascular reactivity, corroborating previous findings [88–90]. Notably, a larger proportion of symptomatic CAS patients exhibited incomplete CoW anatomy, which potentially contributed to their more severe clinical presentation. Contrary to our initial hypothesis, no significant differences were observed in autoregulatory function or inter-hemispheric blood flow between symptomatic and asymptomatic CAS patients. These findings suggest that impaired autoregulation of cerebral blood flow and inefficient inter-hemispheric blood flow may not be major contributing factors to the symptomatic manifestations of CAS in older adults.

In cases of ICA stenosis, cerebral infarction can occur due to both hemodynamic hypoperfusion and embolization. Various clinical and imaging features have been identified as risk factors for late cerebral infarction in asymptomatic ICA stenosis patients [91]. These features include stenosis progression, silent infarction on CT, contralateral transient ischemic attack (TIA) or stroke, intra-plaque hemorrhage on MRI, impaired cerebrovascular reactivity, and spontaneous embolization on TCD [92–101]. These findings underscore the importance of accurately categorizing atherosclerotic ICA stenosis to enhance outcome prediction using novel or repurposed examination methods.

Current international guidelines for the treatment of atherosclerotic CAS consider the degree of stenosis, previous ischemic events (cerebral infarction or TIA), and the overall condition of the patient [91]. Conservative therapy is recommended for CAS that is not hemodynamically significant (less than 70%), while invasive procedures are advised for symptomatic CAS with hemodynamic significance (greater than 70%) [79, 102]. Carotid endarterectomy (CEA) has been shown to effectively prevent secondary events in cases of symptomatic significant (>70%) ICA stenosis [91]. In asymptomatic patients with significant ICA stenosis, CEA can reduce the long-term risk of ischemic events by 50%. However, the perioperative risk of ischemic events is reported to be twice as high in asymptomatic patients (6%) compared to symptomatic patients (3%) [103].

In our study, we did not find significant differences in postoperative complications and surgical outcomes between the groups. The occurrence of adverse effects and postoperative complications was minimal, suggesting that the surgical outcomes were predominantly determined by the quality of the surgery itself. Therefore, the preoperative hemodynamic measurements we recorded had limited predictive value for surgical outcomes in this particular cohort. These findings are consistent with previous studies, emphasizing the critical role of surgical skill and experience in achieving successful outcomes for patients undergoing CAS surgery. Nonetheless, preoperative hemodynamics may still have relevance in guiding the choice of surgical approach.

Several TCD methods are available for evaluating cerebrovascular reactivity, including the hyperventilation test, apnea test, acetazolamide test, and thigh cuff test [66, 67, 104–106]. However, the CCC test stands out as the only functional TCD test that mimics preoperatively the hemodynamic environment similar to clamping during carotid endarterectomy (CEA). Thus, this test has the potential to impact the choice of carotid reconstruction technique. According to previous research, the CCC test has a preoperative predictive value of 66.7% for a positive outcome, 100% for a negative outcome, 100% sensitivity, and 97.8% specificity in predicting the need for a shunt during CEA [107]. In another study with a larger sample size and a broader population of patients with CAS, the CCC test was reported to have a 60% likelihood of indicating the need for intraoperative shunt use [72, 107]. To strengthen this indication, the CCC test can be combined with other functional TCD tests [72, 107].

In this study, we conducted a comprehensive analysis of the amplitude and time variables of blood flow velocity response in the middle cerebral artery (MCA) following the release of carotid compression challenge (CCC). Our observations revealed a unique pattern, characterized by the presence of two peaks in blood flow velocity response after CCC release, in a subset of patients with carotid artery stenosis (CAS). This pattern, referred to as delayed transient hyperemic response (DTHR), was observed in nearly half (48%) of CAS patients, indicating a delayed peak in blood flow during the hyperemic phase. We believe that this phenomenon may be influenced by multifaceted mechanisms involving alterations in local autoregulatory mechanisms, such as myogenic constriction and flow-induced vasoregulation. Thus, we further investigated whether the presence of this late blood flow velocity peak was associated with specific patient characteristics or correlated with static and dynamic blood flow velocity changes.

While we did not observe differences in maximal blood flow evoked by CCC, our findings demonstrated that patients with delayed THRR had lower maximal THRR values. Delayed THRR is characterized by a prolonged reduction in blood flow velocity, indicated by a longer return to baseline (RTB), which may be suggestive of impaired cerebral vascular reactivity. Our results indicate that a significant immediate increase in MCA blood flow velocity after carotid compression cessation, reflected by a high transient hyperemic response ratio, is often accompanied by a rapid induction of vasoconstriction in response to increased flow, as quantified by the rate of blood flow velocity reduction time (RTB). We propose that RTB can serve as a reliable marker of vasoreactivity. Notably, our correlation analysis revealed that prolongation of RTB was associated with decreases in vasoreactivity, as evidenced by delayed or absent transient hyperemia. A smaller increase in blood flow velocity suggests impaired cerebral vasoreactivity, while a prolonged RTB may indicate compensation for the less efficient restoration of blood flow following CCC. In our study, impaired vasoreactivity was associated with longer RTBs. These findings highlight the potential role of RTB as an indicator of impaired vasoreactivity and suggest its utility in assessing cerebrovascular function.

More than half of the patients included in our study experienced a decrease in both heart rate and arterial blood pressure during the CCC maneuver. This observation suggests that a reduction in systemic arterial blood pressure during CCC may contribute to the lower THRR observed after release of the compression cuff. We hypothesize that the normalization of systemic arterial pressure can lead to an additional increase in blood flow, contributing to the delayed transient hyperemia phenomenon. Considering the changes in arterial blood pressure during CCC, we also calculated cerebrovascular resistance (CVR) values. Typically, CVR values at the end of the CCC test, reflecting maximal vasodilation, are low, indicating a well-functioning autoregulation. However, our study’s CVR data revealed reduced autoregulatory capacity in the group exhibiting delayed THR reactions. Autonomic dysregulation may contribute to the observed alterations in heart rate and arterial blood pressure during the CCC test. Previous research has demonstrated a higher prevalence of autonomic dysfunction in patients with carotid atherosclerosis, which has been associated with increased mortality in individuals with cerebrovascular disease [108, 109]. This phenomenon is believed to occur due to decreased carotid sinus distensibility, resulting in reduced baroreceptor sensitivity and abnormal cardiovascular regulatory responses [110]. Clinical trials have confirmed that patients with carotid atherosclerosis exhibit more severe autonomic dysfunction compared to elderly control groups and other individuals with cerebrovascular disease [108]. These findings highlight the importance of monitoring arterial blood pressure concurrently with the assessment of cerebral vascular reactivity.

Furthermore, we found a significant correlation between patient age and decreased amplitude, delayed THR, and prolonged RTB following CCC. Additionally, there was a correlation between age and eGFR values, as well as a higher occurrence of significant drops in blood pressure during CCC in older patients. These results support the detrimental effects of aging on both cerebral and systemic microcirculation and cardiovascular homeostasis, which likely contribute to impaired cerebral vascular reactivity and dysregulation of cerebral blood flow. The cellular and molecular mechanisms underlying cerebrovascular aging and accelerated cerebrovascular aging associated with atherosclerotic diseases are multifaceted, involving dysregulation of calcium signaling pathways and myogenic machinery in smooth muscle cells, endothelial dysfunction, alterations in the extracellular matrix, heightened inflammatory status of the vascular wall, and structural remodeling and rarefaction of cerebral microvessels [39–41, 43, 82, 111–115].

The CoW serves as a crucial network connecting the internal carotid arteries to the vertebral arteries, supplying collateral blood flow to the brain. In patients with CAS, where blood flow through the affected carotid artery is compromised, an intact CoW can play a vital role in maintaining sufficient blood supply to the brain by providing an alternative pathway for blood to reach the affected hemisphere. This collateral circulation helps prevent cognitive dysfunction caused by ischemia and reduces the risk and severity of ischemic strokes. Remarkably, our study found that a larger proportion of symptomatic CAS patients exhibited compromised CoW anatomy compared to asymptomatic CAS patients. This aligns with previous research demonstrating a higher prevalence of absent or hypoplastic segments in the CoW among symptomatic CAS patients [87, 116–119]. In healthy individuals, the prevalence of a complete CoW ranges from 27 to 90%, while in individuals with cerebrovascular disease, it ranges from 18 to 55%. The prevalence of absent or hypoplastic CoW segments can be influenced by various factors, including ethnic and population differences [61, 63, 120].

Variations in the CoW can impact both the temporal and amplitude characteristics of transient hyperemic response and residual cerebral blood flow. Surprisingly, our study did not find significant differences in the amplitude variables of THR or residual CBF during the CCC test between patients with an intact CoW and those with a compromised CoW. However, we did observe variation in delayed THR values between the two patient groups. Our findings demonstrated that patients with CAS and an intact CoW had a lower maximal THRR compared to those with a compromised CoW. These results suggest that the intact CoW may compensate for reduced cerebral blood supply in the affected hemisphere by enhancing inter-hemispheric blood flow. Although the exact mechanisms underlying this compensation are not yet fully understood, it is possible that the intact CoW facilitates increased blood flow through the contralateral hemisphere, leading to improved cerebral perfusion. Another plausible explanation is that compromised CoW could be indicative of more extensive vascular disease, affecting cerebral autoregulation and resulting in a higher maximal THRR. Further research is necessary to explore these hypotheses and elucidate the potential clinical implications of our findings. The unexpected results in our study may be attributed to the heterogeneity within the compromised CoW group. While studies on the anastomosis capacity of the circle of Willis often emphasize the significance of the anterior communicating artery, others emphasize the role of the posterior CoW [121, 122]. In our study, we did not analyze the anterior and posterior CoW systems separately, and the presence of contralateral steno-occlusive CAS might also act as a modifying factor. It is possible that our patient group consisted of subgroups with varying degrees of CoW compromise, which could have influenced the study outcomes. Further research with larger sample sizes and more homogeneous participant groups is necessary to validate and expand upon these findings. Accurately characterizing global cerebral hemodynamics also requires assessing the capacity of the secondary cerebral collateral system, including leptomeningeal and extra-intracranial arterial anastomoses [123].

While our study offers valuable insights, it is crucial to acknowledge its limitations. One limitation is the relatively small and selective sample of patients included. However, it is worth noting that the number of patients recruited is comparable to previous studies conducted with TCD [104, 105, 124, 125]. Although efforts were made to include both symptomatic and asymptomatic patients, there was an imbalance in the representation of these groups, with a predominance of male patients. Moreover, certain subgroups of ICA stenosis were excluded from the study due to the absence of insonation windows and exclusion criteria, potentially introducing bias into the results. Additionally, the adequacy of the temporal insonation window can be influenced by age-related changes in bone structure, as reported in the literature [126]. In our study, the higher proportion of inadequate insonation windows compared to previous reports may be attributed to the older age of our patient cohort. It is also possible that the rigorous study protocol outlined in the methods section contributed to these differences. The described study procedure is operator-dependent, as both TCD and the CCC test require a skilled operator for accurate performance and interpretation. Additionally, it is important to note that TCD measures blood flow velocity in large arteries, which may not fully reflect regional differences in blood flow within smaller vessels. The velocity of blood flow and its change in the proximal cerebral vessels measured by TCD without knowing the arterial pressure that maintains perfusion does not in itself reflect the change in blood flow in the relevant brain tissue. Furthermore, patient safety is a concern during the CCC test; however, it is noteworthy that none of the patients in our study reported any complaints during the examinations. Moreover, it is essential to acknowledge that the various calculated indices introduced in this study, characterizing cerebrovascular responses, are not yet widely employed in clinical practice. Nevertheless, we hope that these indices will be adopted by other researchers, enabling them to compare their findings with those presented in our study.

In conclusion, the evaluation of cerebrovascular reactivity presented in this study offers a new perspective on characterizing patients with significant CAS. By analyzing the duration and type of THR during the CCC test, we provide a more complex understanding of cerebral vasoreactivity. This study is the first to comprehensively analyze time variables, CoW anatomy, and changes in blood pressure and heart rate in characterizing the CCC test in older adults with symptomatic and asymptomatic CAS. Further follow-up studies on a larger cohort of older CAS patients are needed to assess additional relevant clinical outcome measures. The mechanisms responsible for the transient hyperemic response after the CCC test are complex and involve several physiological processes, including myogenic autoregulation, metabolic vasodilation, sympathetic nervous system activation, and endothelium-mediated vasomotor responses. Aging and several risk factors that promote accelerated cardiovascular aging (e.g., hypertension, diabetes mellitus, smoking, hyperlipidemia) can impact the function and phenotype of vascular smooth muscle cells, pericytes, and endothelial cells and the regulation of the tone of resistance arteries, arterioles, and capillaries by mediators released from astrocytes, neurons and perivascular microglia, and immunocytes. Further mechanistic studies are needed to determine how alterations in these synergistic vasoregulatory mechanisms induced by cardiovascular risk factors affect the components of the THR and inter-hemispheric blood flow in older adults and patients with accelerated biological age [127].
